# Marine tardigrades (Heterotardigrada) from Senghor Seamount, Eastern Tropical Atlantic, with the description of two new species from the taxon Stygarctidae

**DOI:** 10.3897/zookeys.1295.205902

**Published:** 2026-10-27

**Authors:** Roman Ruslanovych Trokhymchuk, Jesper Guldberg Hansen, Uliana Pysarenko, Kateryna Subota, Alexander Kieneke

**Affiliations:** 1 Naturalis Biodiversity Center, Darwinweg 2, 2333 CR, Leiden, Netherlands Naturalis Biodiversity Center Leiden Netherlands; 2 German Centre for Marine Biodiversity Research, Senckenberg am Meer, Südstrand 44, 26382, Wilhelmshaven, Germany German Centre for Marine Biodiversity Research, Senckenberg am Meer Wilhelmshaven Germany; 3 Natural History Museum of Denmark, University of Copenhagen, Universitetsparken 15, 2100, Copenhagen, Denmark Natural History Museum of Denmark, University of Copenhagen Copenhagen Denmark; 4 V.N. Karazin Kharkiv National University, Svobody Square 4, 61022, Kharkiv, Ukraine V.N. Karazin Kharkiv National University Kharkiv Ukraine

**Keywords:** Biodiversity, Cabo Verde, Cape Verde, *
Megastygarctides
*, meiobenthos, meiofauna, *
Parastygarctus
*, Tardigrada, taxonomy

## Abstract

The Atlantic Ocean’s seamounts are known for their high diversity of meiofauna. In this study an overview of the marine tardigrade fauna on the yet not studied Senghor Seamount is presented. The materials were collected during the M79/3 cruise of R/V Meteor in the Cabo Verde (Cape Verde) region from Senghor Seamount and a deep-sea reference site. Samples from the seamount yielded 20 taxa: three determined species (*Chrysoarctus briandi*, *Quisarctus armatus*, *Raiarctus colurus*), 15 other taxa (*Angursa* sp., *Batillipes* sp., Halechiniscidae sp., Halechiniscidae gen. A sp. A, *Chrysoarctus* sp., *Dipodarctus aff. subterraneus*, *Dipodarctus* sp., Florarctinae sp., *Halechiniscus cf. perfectus*, *Halechiniscus* sp., Styraconyxidae sp., *Raiarctus aff. variabilis*, *Raiarctus* sp., *Tanarctus* sp., Tholoarctus cf. natans natans), and two new species, for which we provide morphological descriptions: *Megastygarctides hekatae***sp. nov**. with rare genital spines; and *Parastygarctus potercha***sp. nov**. with a paedomorphic appearance. In contrast, the deep-sea reference station is represented only by two taxa (*Angursa abyssalis* and *Angursa* sp.). This observation supports the hypothesis of seamounts as hotspot habitats for marine tardigrades.

## Introduction

Seamounts are elevations of the sea floor, typically of volcanic origin (Staudigel and Clague 2010), and some exhibit relatively shallow summits and deep-sea bases (Yesson et al. 2011). These landforms significantly influence the shaping of fauna due to alterations in currents dynamics around them, and a significant contribution of organic matter (Mohn et al. 2021; Zhao et al. 2023). Seamounts, particularly those in the Atlantic Ocean, are considered hotspots (oases) for meiofauna, usually characterised by its abundance, biodiversity, and, primarily, the presence of endemic taxa compared to the surrounding deep-sea areas (George and Schminke 2002; Büntzow 2011; Zeppilli et al. 2013; George et al. 2021; Kieneke et al. 2025; George et al. 2026). Some tardigrade surveys have already been conducted on seamounts of the Atlantic Ocean (Faroe Bank, Condor Seamount, Great Meteor Seamount, and Princess Alice Bank) and have yielded quite a number of species records and newly described marine species (Hansen et al. 2001; Hansen et al. 2012; Hansen and Kristensen 2021; Kristensen et al. 2015; Tchesunov 2018; Trokhymchuk et al. 2025). Especially the studies on the northeast Atlantic Faroe Bank demonstrated a high level of tardigrade biodiversity, despite their generally low abundance in deep-sea sediments, suggesting habitats of seamounts are supporting diverse and unique tardigrade communities (Jørgensen and Kristensen 2001; Hansen 2005, 2007; Hansen and Kristensen 2020; Hansen et al. 2001, 2003). In order to test this hypothesis, further seamounts need to be explored with a high taxonomic resolution at the species level. In this study, we examine the tardigrade fauna of the Senghor Seamount (SSM), Cabo Verde archipelago, and describe two new species of armoured marine tardigrades (Stygarctidae): *Megastygarctides hekatae* sp. nov. and *Parastygarctus potercha* sp. nov. From comparing the tardigrade community of the seamount with that of the deep-sea reference site, we expect to observe differences in both biodiversity and abundance, with the highest values occurring on the seamount as can be deduced from previous studies (Trokhymchuk and Kieneke 2024; Kieneke et al. 2025). In addition, dichotomous and pictorial identification keys to the species of *Megastygarctides* and *Parastygarctus* were constructed.

## Material and methods

### Sampling and processing

**Figure 1.**
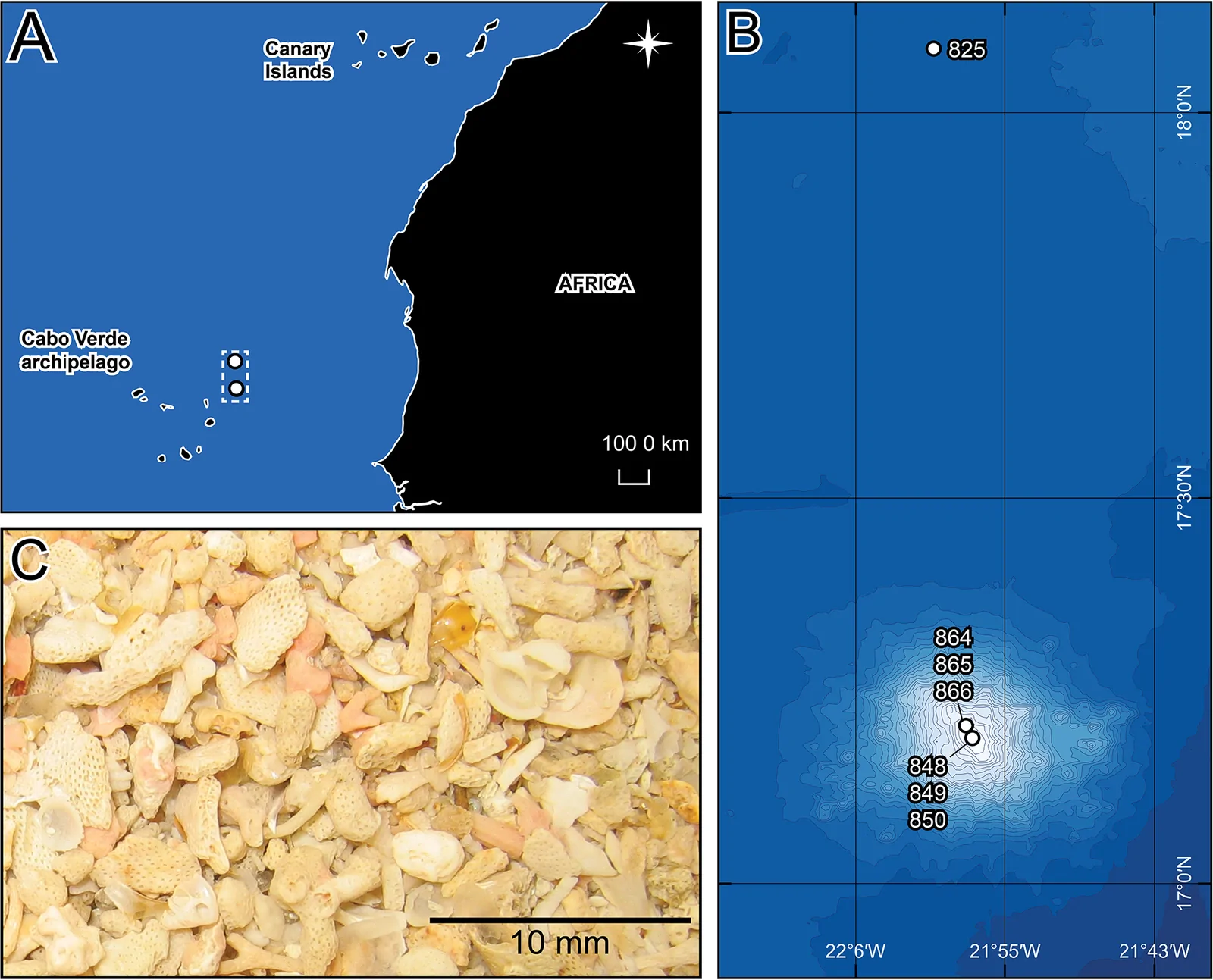
Study area during the M79/3 expedition of R/V Meteor (September 2009). **A**. Map covering the Cape Verde archipelago, Canary Islands, western Africa and sampling stations (indicated by white points); **B**. Study area covering Senghor Seamount and northern reference sampling site 825 (the shown area is indicated by dashed rectangle in Fig. 1A); **C**. Biogenic coarse sand from Senghor Seamount summit, station 845 (not in this study) at 102.9 m depth. Map from GEBCO.

Sediment samples were collected with a multiple corer (MUC) during the R/V Meteor cruise M79/3 in the Cabo Verde (Cape Verde) region (Fig. 1), in September 2009 (Christiansen et al. 2011; Kieneke et al. 2025). Samples investigated in this study were taken from the summit plateau of SSM and from one of two deep-sea reference sites, the analysed one being approximately 100 km to the north of the seamount (Fig. 1A, B, Table 1). The summit sediment is composed mainly of coarse carbonate material of organic origin, such as fragments of corals, echinoderms, bryozoans, and molluscs (Fig. 1C). Reference site sediment is mainly composed of fine ooze (Christiansen et al. 2011).

**Table 1.** Sampling sites investigated in this study.

**Cruise**	**Station**	**Corer**	**Latitude (N), Longitude (E)**	**Depth [m]**	**region**
M79-3	825	7	18°05.00'N, 22°00.20'W	3293.9	deep reference N
M79-3	848	5	17°11.31'N, 21°57.20'W	101.3	summit central
M79-3	849	2	17°11.31'N, 21°57.20'W	102.0	summit central
M79-3	850	2	17°11.31'N, 21°57.20'W	102.4	summit central
M79-3	864	2	17°12.29'N, 21°57.69'W	132.4	summit NW
M79-3	864	7	17°12.29'N, 21°57.69'W	132.4	summit NW
M79-3	865	4	17°12.30'N, 21°57.70'W	383.2	summit NW
M79-3	866	3	17°12.30'N, 21°57.70'W	133.6	summit NW

The upper 5 cm of sediments were cut from each core sample and immediately fixed with formaldehyde at the final concentration of approximately 8% (v/v). Meiofaunal specimens were extracted by density gradient centrifugation (Somerfield et al. 2005) using the colloidal silica Levasil®. After centrifugation, the supernatant liquid containing the meiofauna was poured over a sieve of 40 µm mesh size. In order to facilitate sorting, the centrifuged samples were stained with Rose Bengal. The meiofauna was sorted at the department DZMB of Senckenberg am Meer, Germany, using Leica M80 and M125 stereo microscopes.

### Microscopy and imaging

Permanent slides of tardigrades were prepared with a well-established technique using beeswax-paraffin and glycerol (Trokhymchuk and Kieneke 2025; Trokhymchuk et al. 2025). Light microscopy examinations were conducted with an Olympus BX53 upright research microscope equipped with differential interference contrast (DIC) and an intermediate tube with interchangeable magnifications. Microphotographs were made with a digital microscope camera Euromex HD-Ultra VC.3036-HDS. Preparations for scanning electron microscopy (SEM) followed the methods as described in Trokhymchuk and Kieneke (2025), Trokhymchuk et al. (2024), Minowa et al. (2025). SEM was carried out with a TESCAN VEGA3 SEM operated with 10 kV acceleration tension.

Tardigrades were measured during light microscopic examination, and only if the measured feature was undamaged. All the cirri were measured, starting from the cirrophore and terminating at the end tip of the flagellum. Digital drawings were made in the Procreate app. Stacked images were created in PICOLAY V2020-08-13 by Heribert Cypionka (www.picolay.de). Figures were assembled in Adobe Photoshop and Adobe InDesign. The map was made with open-source software QGIS 3.40 Bratislava. Genera were determined according to Fontoura et al. (2017), and Hansen and Kristensen (2020). Species were identified according to the original descriptions (see Results and Discussion for references). The validity of species followed Guidetti and Bertolani (2005), Degma and Guidetti (2007), and Degma and Guidetti (2026). We use the open nomenclature “aff.” to denote that a presumptive new species belongs to a species complex related with the mentioned species; “cf.” in cases of uncertainties in determination due to poor preserved individuals; and “sp.” when individuals cannot be related as any of known species (Bengtson 1988). Terminology used in the species descriptions according to Hansen and Kristensen (2006), Hansen et al. (2012), and Hansen and Kristensen (2020).

### Collection

All specimens (slides and SEM stubs) are deposited in the Tardigrada collection of the Senckenberg Natural History Museum, Frankfurt am Main, Germany (**SMF**), under the inventory numbers SMF 1386–1604 (accessible via https://search.senckenberg.de/aquila-public-search/search). All the occurrences are additionally submitted to GBIF (Senckenberg Collection TardigradaSMF. Occurrence dataset accessible via https://doi.org/10.15468/uvr84c).

## Results

### Marine tardigrade fauna

**Figure 2.**
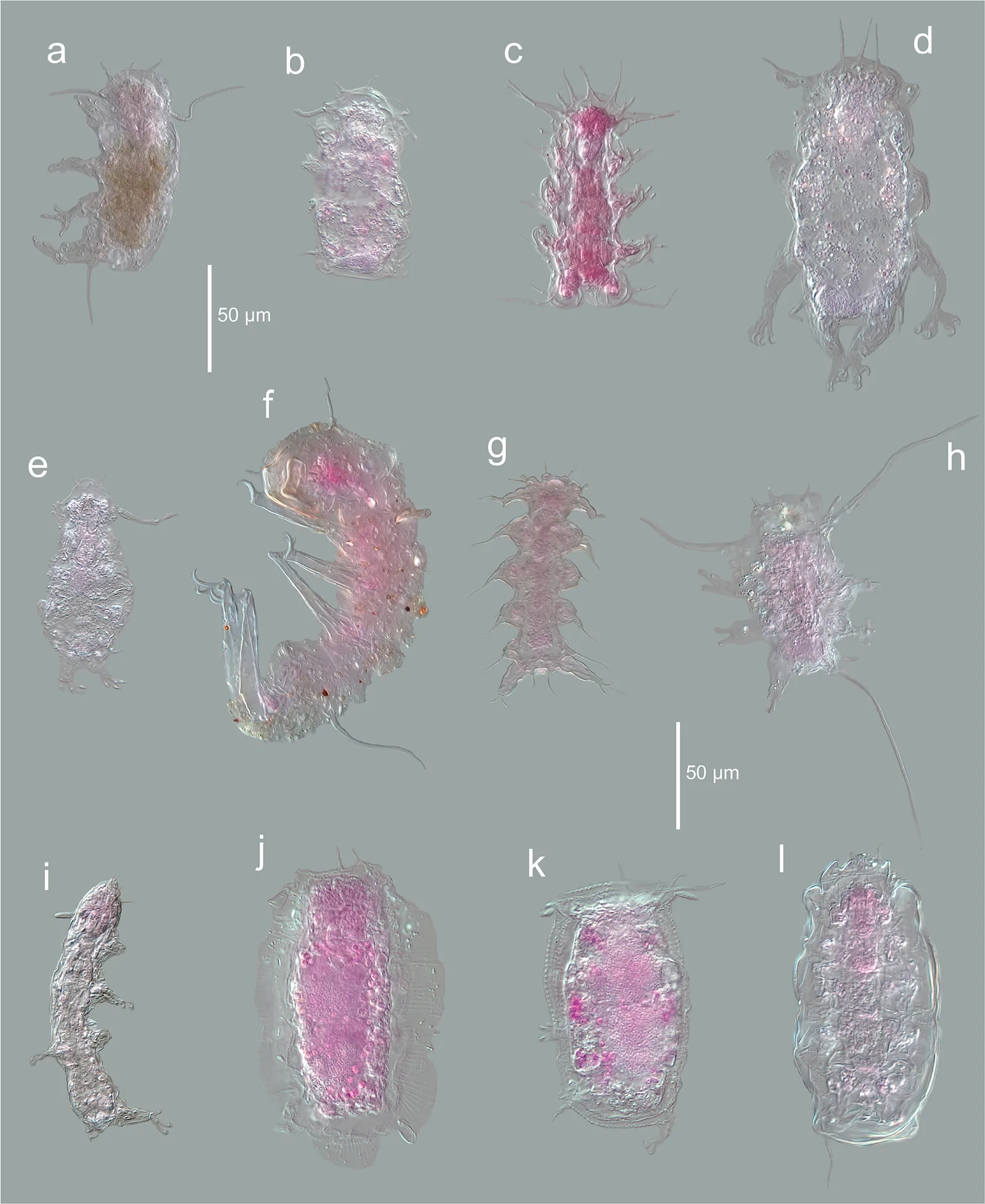
Selected marine tardigrade representatives from this study. Halechiniscidae (**a**–**e**): **a**. *Dipodarctus aff. subterraneus*; **b**. *Chrysoarctus briandi*; **c**. Halechiniscidae gen. A sp. A; **d**. *Halechiniscus cf. perfectus*; **e**. *Quisarctus armatus*; Stygarctidae (**f**, **g**): **f**. *Megastygarctides hekatae* sp. nov.; **g**. *Parastygarctus potercha* sp. nov.; Tanarctidae (**h**): **h**. *Tanarctus* sp.; Styraconyxidae (**i**–**l**): **i**. *Angursa abyssalis*; **j**. *Raiarctus colurus*; **k**. *Raiarctus aff. variabilis***l**. Tholoarctus cf. natans natans. Specimens a–h, j–l from Senghor Seamount. Specimen i from deep-sea reference site. All specimens to the same scale. Pink colour from Rose Bengal staining.

In total, 219 marine tardigrade individuals were recovered and analysed from the eight selected samples. For the SSM summit we reported 212 tardigrade specimens, three of them (station 865, core 4) were deteriorated so we could only identify them as Heterotardigrada. In total, the tardigrade fauna of SSM consists of 20 taxa. Batillipedidae constitutes 0.5% and is represented by *Batillipes* sp. Halechiniscidae is the most abundant family, covering 52.6%, and represented by unidentified Halechiniscidae, *Dipodarctus* sp., *Dipodarctus aff. subterraneus* (*Dipodarctus subterraneus* (Renaud-Debyser, 1959)) (Fig. 2a), representatives of Florarctinae, *Chrysoarctus* sp., *Chrysoarctus briandi* Renaud-Mornant, 1984 (Fig. 2b), Halechiniscidae gen. A sp. A (Fig. 2c), *Halechiniscus cf. perfectus* (*Halechiniscus perfectus* Schulz, 1955) (Fig. 2d), and *Quisarctus armatus* Trokhymchuk, Arbizu, Kristensen & Kieneke, 2025 (Fig. 2e). Stygarctidae comprises 6.7% and is represented by the two new species *Megastygarctides hekatae* sp. nov. (Fig. 2f) and *Parastygarctus potercha* sp. nov. (Fig. 2g) described in this publication. Styraconyxidae constitutes 26.3%, and is represented by unidentified representatives of Styraconyxidae, *Angursa* sp., *Raiarctus* sp., *Raiarctus colurus* Renaud-Mornant,1981 (Fig. 2j), *Raiarctus aff. variabilis* (*Raiarctus variabilis* D’Addabbo Gallo, Grimaldi de Zio & Morone de Lucia, 1986) (Fig. 2k), and Tholoarctus cf. natans natans (*Tholoarctus natans natans* Kristensen & Renaud-Mornant, 1983) (Fig. 2l). Tanarctidae covers 13.9% and is represented by *Tanarctus* sp. (Fig. 2h).

From the deep-sea reference area, we identified two taxa: six specimens of *Angursa abyssalis* Renaud-Mornant, 1981 (Fig. 2i), and a single *Angursa* sp. The tardigrade abundances in samples are provided in Table 2. The representatives of Florarctinae, *Angursa* sp., *Dipodarctus aff. subterraneus*, *Raiarctus aff. variabilis*, and *Tanarctus* sp. are possibly new, undescribed species. However, the material was not in a proper state for making reliable species descriptions.

**Table 2.** Marine tardigrades taxa list with their abundances. Three deteriorated specimens from 865-4 not in the list.

**Station code**	**825-7**	**848-5**	**849-2**	**850-2**	**864-2**	**864-7**	**865-4**	**866-3**
**Depth (m)**	**3293.9**	**101.3**	**102.0**	**102.4**	**132.4**	**132.4**	**383.2**	**133.6**
**Region**	**deep reference N**	**summit central**	**summit central**	**summit central**	**summit NW**	**summit NW**	**summit NW**	**summit NW**
** Batillipedidae **								
*Batillipes* sp.					1			
** Halechiniscidae **								
Halechiniscidae sp.				6			6	
Halechiniscidae gen. A sp. A		10	5	4	11	5		12
** Dipodarctinae **								
*Dipodarctus* sp.				3				
*Dipodarctus aff. subterraneus*				1				
** Florarctinae **								
Florarctinae sp.			1	2		1		
** Halechiniscinae **								
*Chrysoarctus* sp.			1	5		1		4
*Chrysoarctus briandi*		1		1				4
*Halechiniscus* sp.			1		1		7	
*Halechiniscus cf. perfectus*								4
** Quisarctinae **								
*Quisarctus armatus*		3	2	8				
** Stygarctidae **								
** Megastygarctidinae **								
*Megastygarctides hekatae* sp. nov.				2				
** Stygarctinae **								
*Parastygarctus potercha* sp. nov.		1	1	10				
** Styraconyxidae **								
Styraconyxidae sp.			1		1	2	1	1
*Angursa* sp.	1			1				3
*Angursa abyssalis*	6							
*Raiarctus* sp.				2	1	1		1
*Raiarctus colurus*		5	9	9	4	3		1
*Raiarctus variabilis*		1	3	4				
Tholoarctus cf. natans natans				1				
** Tanarctidae **								
*Tanarctus* sp.		5	5	9	2		1	7
**Total abundance**	**7**	**26**	**29**	**68**	**21**	**13**	**15**	**37**

### Taxonomy

#### Tardigrada Doyère, 1840

**Heterotardigrada Marcus, 1927**

**Stygarctidae Schulz, 1951**

**Megastygarctidinae Bello & Grimaldi de Zio, 1998**

***Megastygarctides* McKirdy, Schmidt & McGinty-Bayly, 1976**

##### Megastygarctides hekatae sp. nov.

Taxon classificationAnimaliaArthrotardigradaStygarctidae

64616E13-DE49-5DC7-9074-8877E6174B58

https://zoobank.org/45A74904-875A-4719-B07F-FC083477C0C9

**Figure 3.**
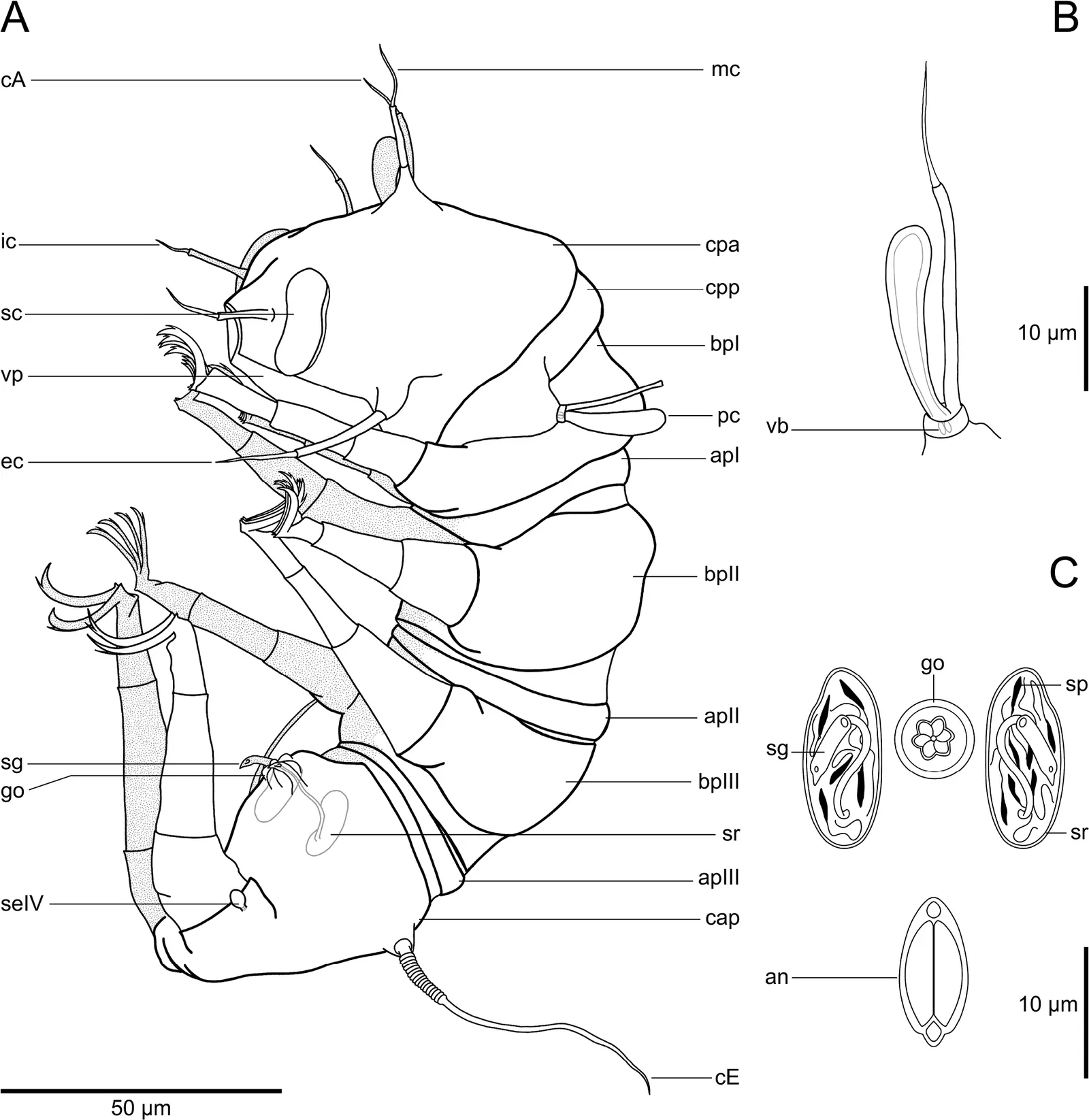
Drawings of *Megastygarctides hekatae* sp. nov. **A**. Holotype female (SMF 1484), ventrolateral view, structures from the right body side shaded; **B**. Holotype female (SMF 1484); reconstruction of primary clava and cirrus *A* on the common cirrophorus with van der Land’s body; **C**. paratype female (SMF 1482) reconstruction of genital structures. Abbreviations: an—anus, apI–apIII—accessory plates I–III, bpI–bpIII—dorsal body plate I–III, cA—cirrus *A*, cE—cirrus *E*, cap—caudal plate, cpa—anterior cephalic plate, cpp—posterior cephalic plate, ec—external cirrus, go—female gonopore, ic—internal cirrus, mc—median cirrus, pc—primary clava, seIV—leg IV sensory organ, sc—secondary clava, sg—genital spine, sp—spermatozoan, sr—seminal receptacle (internal), vb—van der Land’s body, vp—ventral cephalic plate.

**Figure 4.**
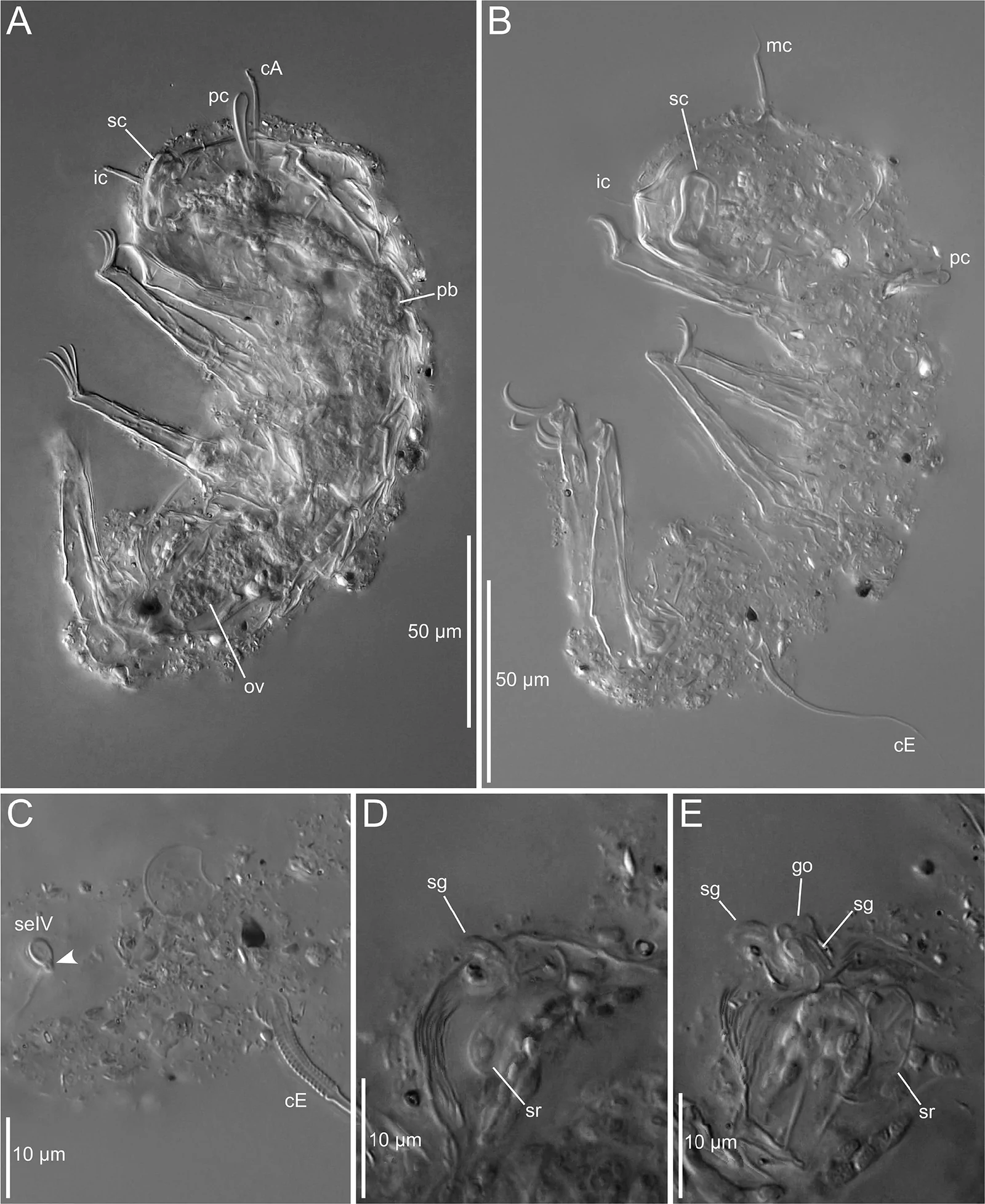
DIC microphotographs of *Megastygarctides hekatae* sp. nov. holotype female (SMF 1484). **A**. Habitus, dorsolateral view; **B**. Habitus, ventrolateral view; **C**. Caudal region, base of leg IV, note van der Land’s body (arrowhead); **D**. Caudal region, reproductive organs; **E**. Caudal region, gonopore and genital spines. Abbreviations: cA—cirrus *A*, cE—cirrus *E*, go—female gonopore, ic—internal cirrus, mc—median cirrus, ov—ovary, pb—pharyngeal bulb, pc—primary clava, seIV—leg IV sensory organ, sc—secondary clava, sg—genital spine, sr—seminal receptacle.

**Figure 5.**
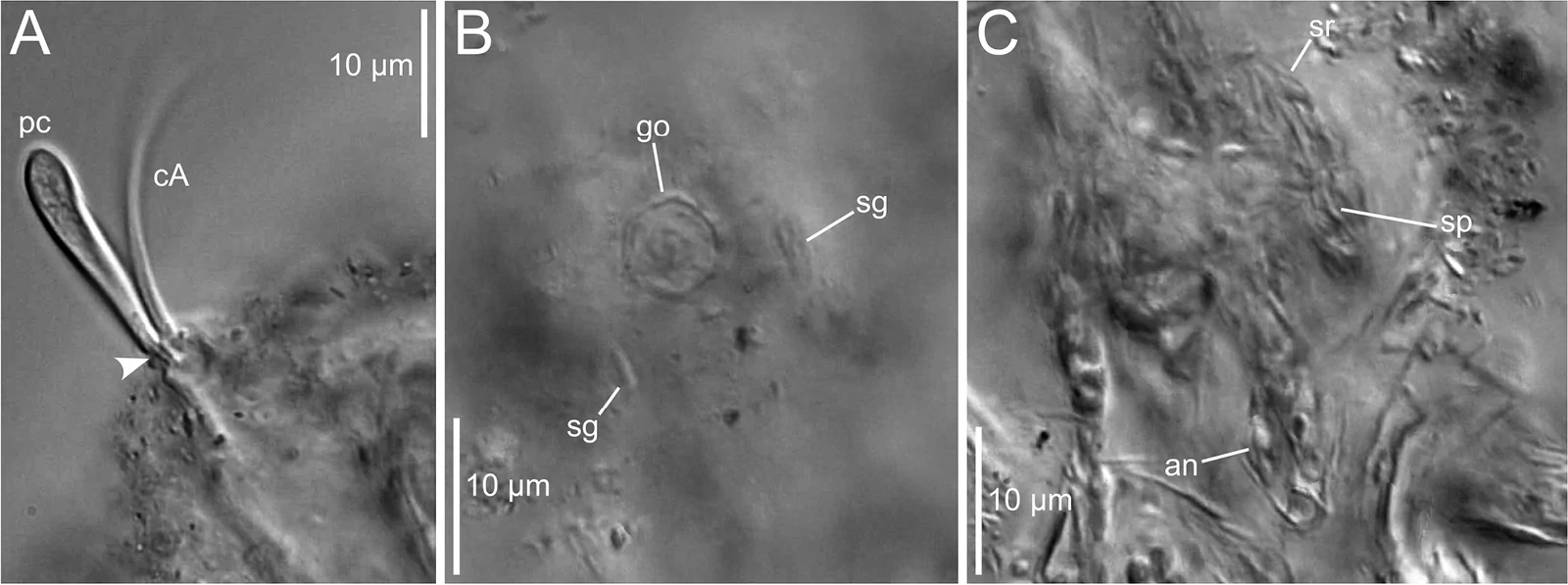
DIC microphotographs (before SEM) of *Megastygarctides hekatae* sp. nov. paratype female (SMF 1482). **A**. Cephalic region, note van der Land’s body (arrowhead); **B**. Caudal region, ventral view, gonopore and genital spines; **C**. Caudal region, ventral view, seminal receptacle, and anus. Abbreviations: an—anus, cA—cirrus *A*, go—female gonopore, pc—primary clava, sg—genital spine, sp—spermatozoan, sr—seminal receptacle.

**Figure 6.**
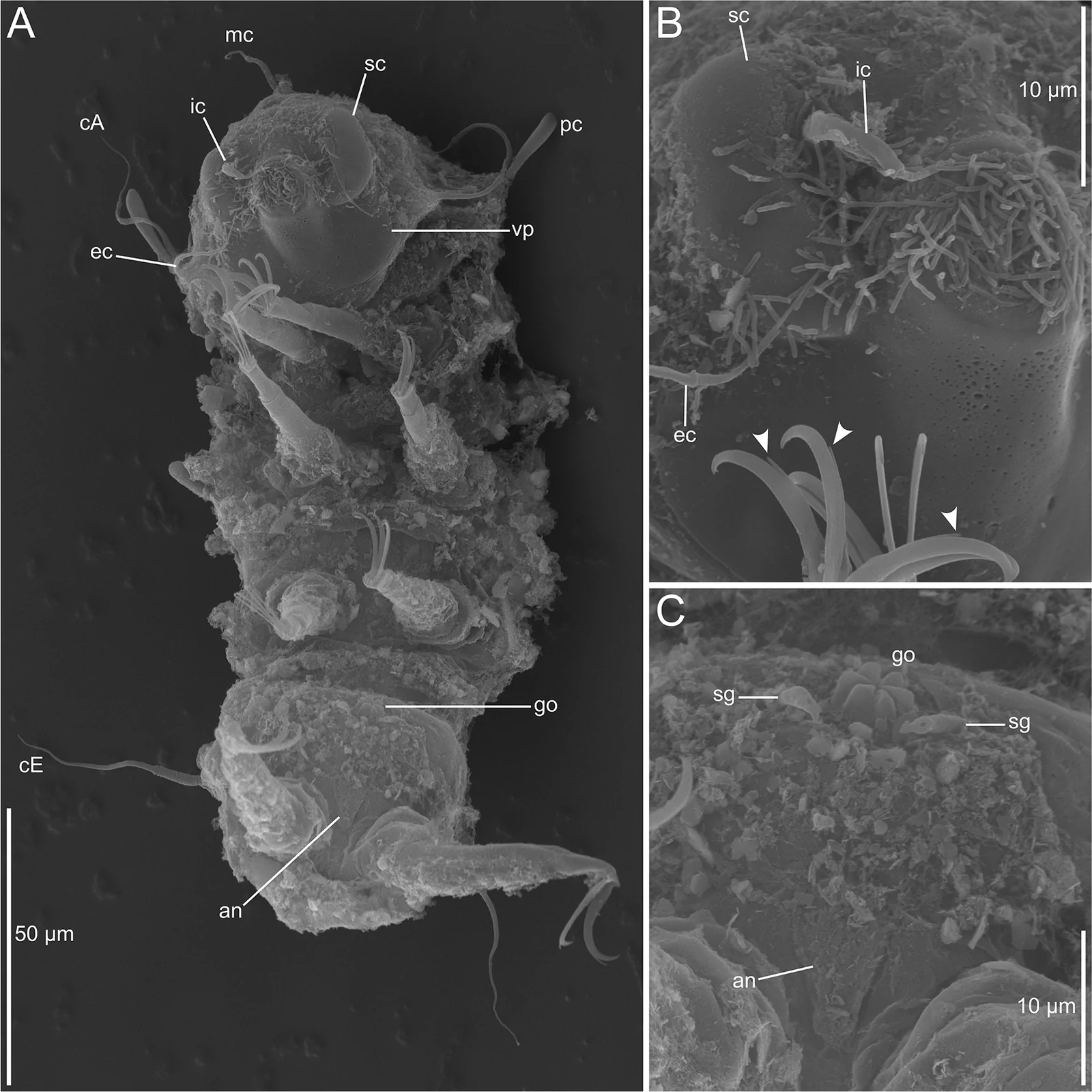
SEM micrographs of *Megastygarctides hekatae* sp. nov. paratype female (SMF 1482). **A**. Habitus, ventral view; **B**. Cephalic region, ventral view, note dorsal spurs of leg I claws (arrowheads); **C**. Caudal region, ventral view. Abbreviations: an—anus, cA—cirrus *A*, cE—cirrus *E*, ec—external cirrus, go—female gonopore, ic—internal cirrus, mc—median cirrus, pc—primary clava, sc—secondary clava, sg—genital spine, vp—ventral cephalic plate.

Figs 3, 4, 5, 6, Table 3

###### Diagnosis.

*Megastygarctides* with three body plates and three accessory plates encircling the body. Cephalic plate is divided into an anterior part and a posterior part. Ventral cephalic plate is present. Primary clavae club-shaped. Secondary clavae dome-shaped. The genital ducts of the two oval seminal receptacles are extended out of the body as two short hollow spines with inferior hooks.

**Table 3.** *Megastygarctides hekatae* sp. nov. measurements of selected morphological structures. SMF 1482 measured before SEM. Numbers are given in micrometres (µm). Abbreviations: na—data not available.

	**Holotype**	**Paratype**
specimen	slide	SEM stub
code	SMF 1484	SMF 1482
sex	female	female
Body length	193.3	185.6
Body width	na	42.3
Median cirrus	28.0	27.9
Internal cirri	23.3	18.9
External cirri	36.3	na
Cirri *A*	28.6	31.8
Primary clavae	17.0	17.9
Secondary clavae	24.5	20.1
Cirri *E*	64.5	61.0
Leg IV sensory organ	3.6	4.3
Leg I claws	16.5	14.4
Leg II claws	14.5	13.9
Leg III claws	14.0	14.0
Leg IV claws	19.5	18.7
Anus--gonopore	13.0	17.0

###### Type locality.

Station 850, 17°11.31'N, 21°57.20'W, 102.4 m b.s.l., Senghor Seamount, summit central, September 2009, biogenic coarse sand, MUC sample. Paratype was collected from the same site.

###### Type material.

***Holotype***: • 1♀ (M79-3-850-2_4v74, SMF 1484) on slide. ***Paratype***: • 1♀ (M79-3-850-2_1v74, SMF 1482) prepared for SEM, on stub.

###### Description.

***Holotype***. Mature female (193.3 µm long) with cuticular armour divided into cephalic plate, three body plates, three accessory plates and caudal plate (Figs 3, 4A, B). The cephalic plate is divided into the anterior and posterior part. On the ventral side of anterior cephalic plate, a ventral cephalic plate is present originating anteriorly from the mouth cone, and ends posteriorly almost at the cephalic plate division. All cephalic cirri arise from a cirrophorus and consist of scapus and flagellum. Internal cirrus is 23.3 µm long. External cirrus is 36.3 µm long and arises from a well-developed cirrophorus. Median cirrus is 28.0 µm long. Elongated club-shaped primary clava (17.0 µm long) and cirrus *A* (28.6 µm long, the other cirrus flagellum is broken) arises from the same lateral head lobe, and a common membrane (cirrophorus) surrounds the base of the primary clava and, with a van der Land’s body inside the base (Fig. 4A, B). Secondary clava is kidney-shaped with a thick cuticle. Mouth cone is retracted. Pharyngeal bulb measures 13.8 μm × 13.0 μm. The other structures of the bucco-pharyngeal apparatus are not visible. Cuticle of body plate is not punctate. Cirrus *E* (64.5 µm long, measured for the whole structure with cirrophorus) arises from cirrophorus and divides into the telescopically inserted annulated scapus and the flagellum (Fig. 4B, C). Sensory organs only on legs IV. Leg IV sensory organ (3.6 µm long) as spherical papilla resting on a short peduncle that contains a van der Land’s body (Fig. 4C). All claws with dorsal spur. Leg IV claws are the longest (19.5 µm long) and the widest, followed by claws on leg I (16.5 µm long) and leg II (14.5 µm long). Leg III claws are the thinnest and with 14.0 µm length the shortest. Rosette-shaped female gonopore present on the ventral side of the caudal plate (Fig. 4E). Seminal receptacles as oval pouches connected by seminal ducts with openings posterior to gonopore and in the form of a hollow spine with short inferior hook (Fig. 4D, E). Single ovary consists of numerous oocytes (Fig. 4A). Anus closed by two large lateral lobes, and small anterior and posterior lobes. Anus is 13.0 µm posteriorly to gonopore.

###### Remarks on paratype.

The single paratype SMF 1482 is another adult female that corresponds to the morphology of the holotype. The primary clavae and cirri *A* cirrophori exhibit better for observation (Fig. 5A). Gonopore, genital spines and anus are present and well observable (Fig. 5B, C). Only a single seminal receptacle filled with spermatozoa (Fig. 5C). The SEM investigation (Fig. 6) helped to observe cephalic structures, such as prominent ventral cephalic plate, claws, and caudal region.

###### Differential diagnosis.

*Megastygarctides hekatae* sp. nov. is most similar to *M. christinae* Hansen & Kristensen, 2006 due to presence of well-developed body plates, ventral cephalic plate and, most importantly, genital spines—unique structures for tardigrades that are only reported for *M. christinae* so far. However, *M. hekatae* sp. nov. can be distinguished from *M. christinae* by the shape of primary clavae that are more elongated in the new species. Hansen and Kristensen (2006) reported male of *M. christinae* with the same form and length of primary clavae as females. Therefore, we consider this trait to be stable for morphological differentiation of species, although we have so far, no male specimen of the new species. Cirri *E* (measured for both species from cirrophorus to the tip end of flagellum) of *M. hekatae* sp. nov. are distinctly longer: 61–64.5 µm compared to 36–43 µm in *M. christinae*. *Megastygarctides hekatae* sp. nov. lacks the second accessory body plate between body plate I and body plate II as present in *M. christinae*. The length of the claws on all limbs of *M. hekatae* sp. nov. is noticeably greater than in *M. christinae*: for leg I 14.4–16.5 vs 9–10 µm, for leg II 13.9–14.5 vs 9–11 µm, for leg III 14 vs 10–12 µm, for leg IV 18.7–19.5 vs 12–13 µm. However, genital spines of *M. hekatae* sp. nov. are shorter than genital spines of *M. christinae*. *Megastygarctides hekatae* sp. nov. has more elongated seminal receptacles and anus.

###### Etymology.

Hekate (Ancient Greek: *Ἑκάτη* derived from ἑ*κάς* (*hekás*, *“*far away*”*) is a Greek mythological goddess often associated with crossroads, borders, the night, and witchcraft. Her name originates from the numeral *hekaton* (Ancient Greek: *ἑκατόν)* meaning “one hundred”, referring to the species type locality depth of approximately 100 meters—the deepest record for all the known *Megastygarctides* spp. so far. We decided to use the species epithet “*hekatae*” instead of the “*hekaton*” because of the tradition of feminine species names in the genus Megastygarctides (e.g., *M. christinae*, *M. gerdae*).

#### Stygarctinae Schulz, 1951

***Parastygarctus* Renaud-Debyser, 1965**

##### Parastygarctus potercha sp. nov.

Taxon classificationAnimaliaArthrotardigradaStygarctidae

95574276-1469-5410-9A8F-CCB5B154F236

https://zoobank.org/6D25DAB0-4D65-419B-8BA9-934973BE3BC6

**Figure 7.**
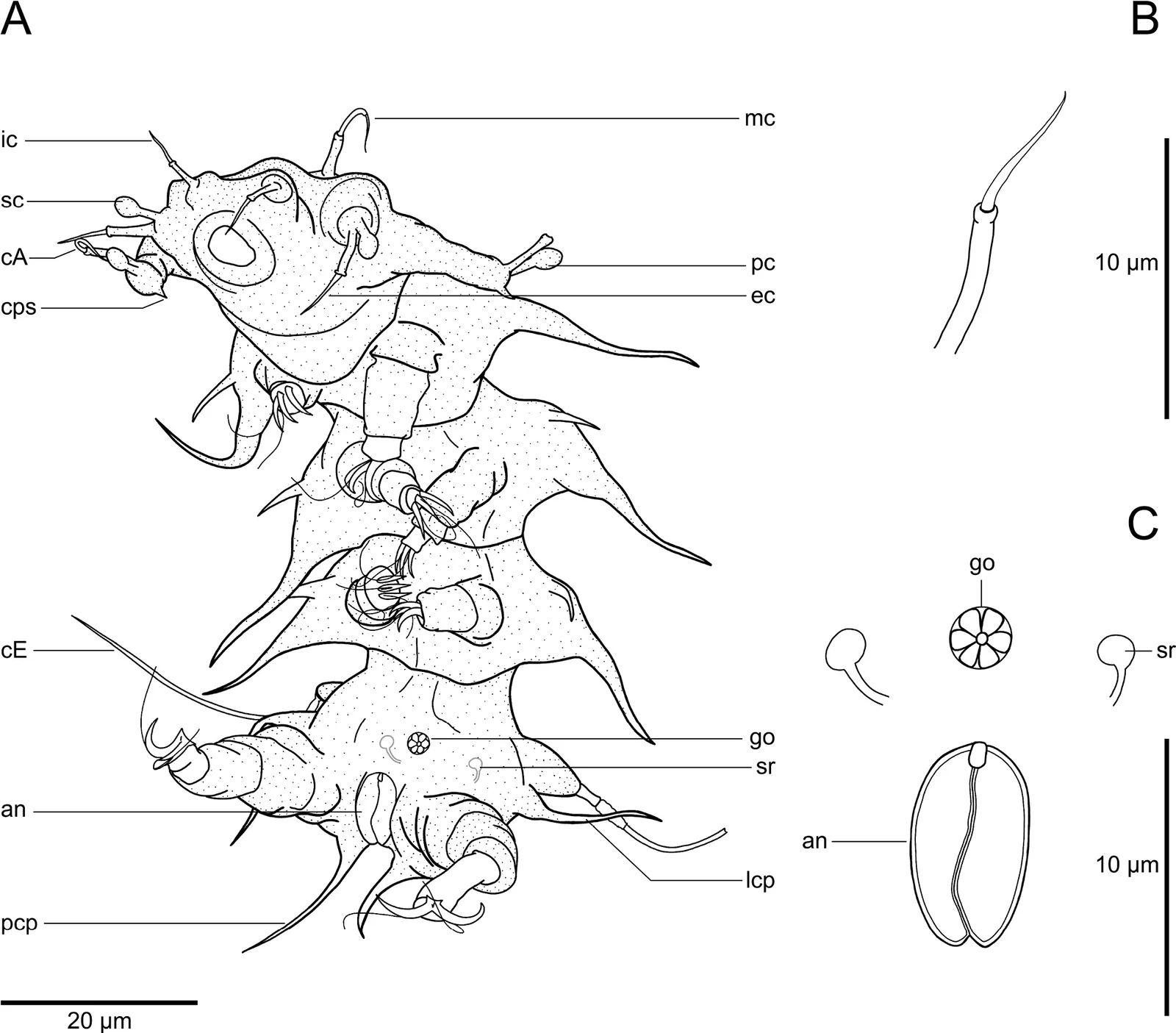
Drawings of *Parastygarctus potercha* sp. nov. holotype female (SMF 1493). **A**. Ventral view; **B**. Cirrus *A* reconstruction; **C**. Reconstruction of genital structures. Abbreviations: an—anus, cA—cirrus *A*, cE—cirrus *E*, cps—cephalic plate lateral lobe posterior spine, ec—external cirrus, go—female gonopore, ic—internal cirrus, lcp—lateral caudal process, mc—median cirrus, pc—primary clava, pcp—posterior caudal process, sc—secondary clava, sr—seminal receptacle (internal).

**Figure 8.**
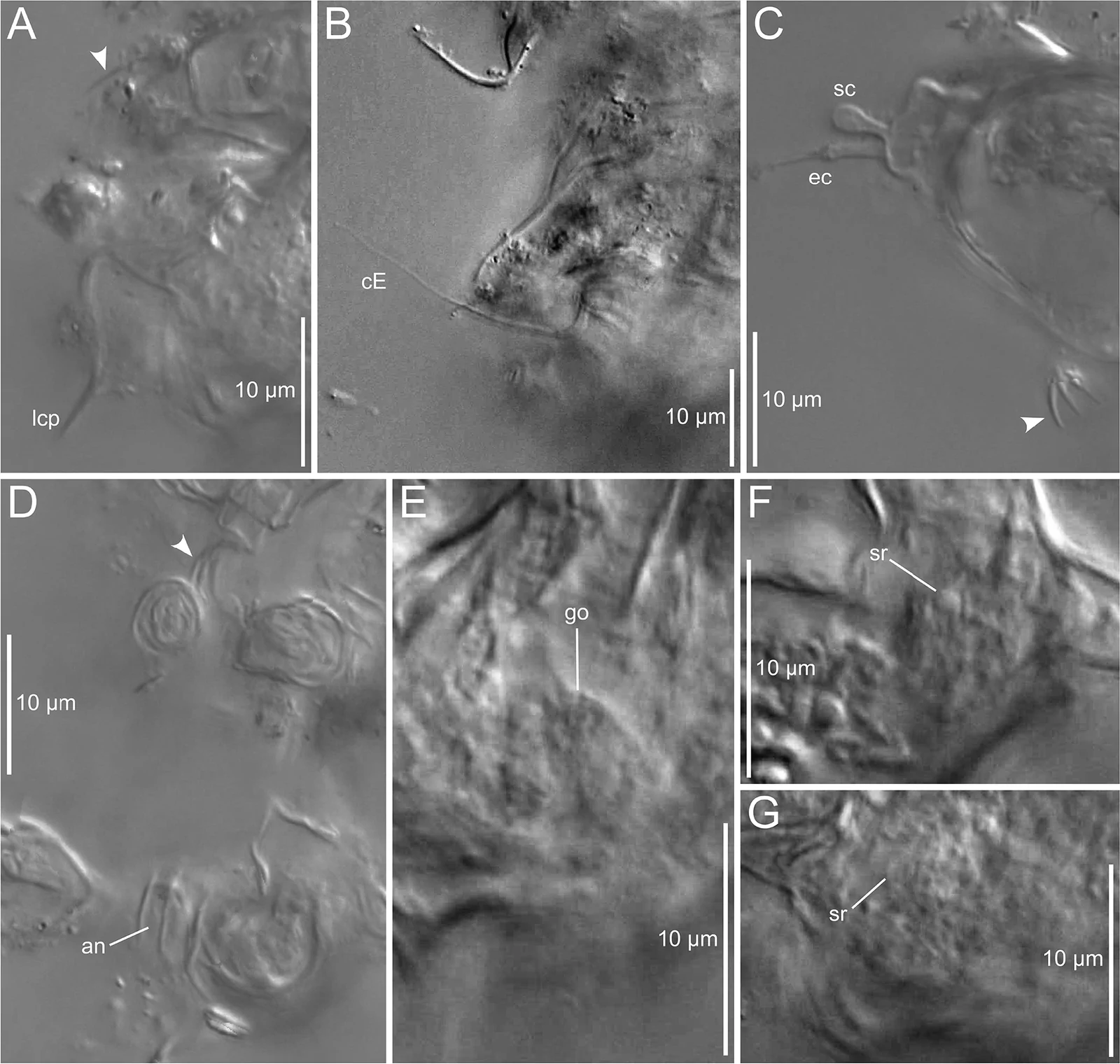
DIC microphotographs of *Parastygarctus potercha* sp. nov. holotype female (SMF 1493). **A**. Caudal region, dorsal view, note anterior lateral process (arrowhead); **B**. Caudal region, dorsal view with cirrus *E*; **C**. cephalic region, note three claws of leg I (arrowhead); **D**. Caudal region, ventral view, note three claws of leg II (arrowhead); **E**. Caudal region, ventral view, gonopore region; **F**. Caudal region, ventral view, seminal receptacle of left side; **G** Caudal region, ventral view, seminal receptacle of right side. Abbreviations: an—anus, cE—cirrus *E*, go—female gonopore, lcp—lateral caudal process, pcp—posterior caudal process, sc—secondary clava, sr—seminal receptacle.

**Figure 9.**
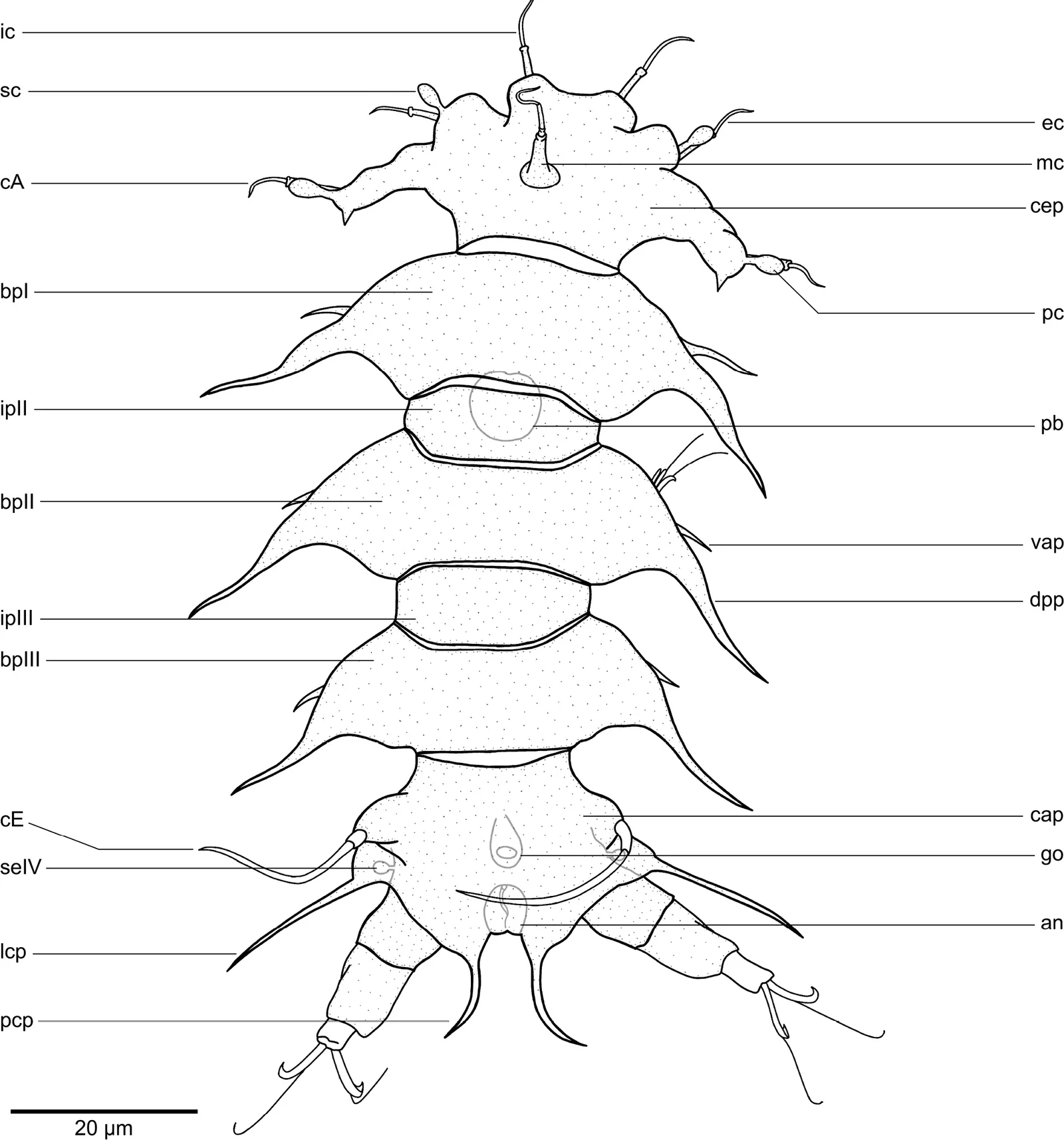
Drawing of *Parastygarctus potercha* sp. nov. paratype male (SMF 1490), dorsal view. Abbreviations: an—anus (ventral), bpI–bpIII—dorsal body plate I–III, cA—cirrus *A*, cE—cirrus *E*, cap—caudal plate, cep—cephalic plate, ec—external cirrus, dpp—dorsal posterior body process, go—male gonopore (ventral), ic—internal cirrus, ipII–ipIII—intersegmental plate II–III, lcp—lateral caudal process, mc—median cirrus, pb—pharyngeal bulb (internal), pc—primary clava, pcp—posterior caudal plate process, sc—secondary clava, seIV—leg IV sensory organ (ventral), vap—ventral anterior body process.

**Figure 10.**
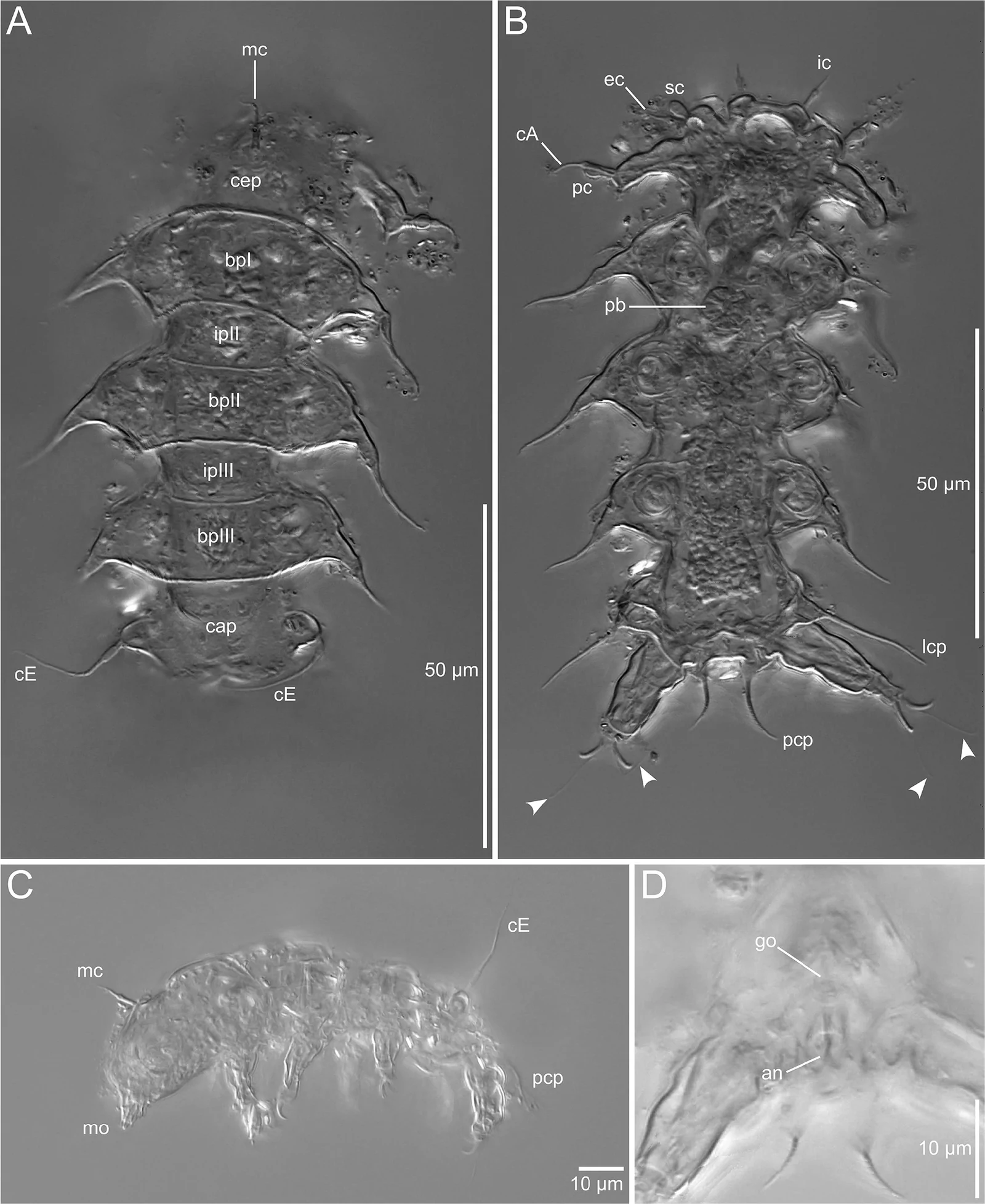
DIC microphotographs of *Parastygarctus potercha* sp. nov. paratype males (A, B, D SMF 1490, C SMF 1604). **A**. Habitus, dorsal view; **B**. Habitus, ventral view, note claw dorsal filament (arrowheads); **C**. Habitus, lateral view; **D**. Caudal region, ventral view with gonopore and anus. Abbreviations: an—anus, bpI–bpIII—dorsal body plate I–III, cA—cirrus *A*, cE—cirrus *E*, cap—caudal plate, cep—cephalic plate, ec—external cirrus, go—male gonopore, ic—internal cirrus, ipII–ipIII—intersegmental plate II–III, lcp—lateral caudal process, mc—median cirrus, mo—mouth cone, pb—pharyngeal bulb, pc—primary clava, pcp—posterior caudal process, sc—secondary clava.

**Figure 11.**
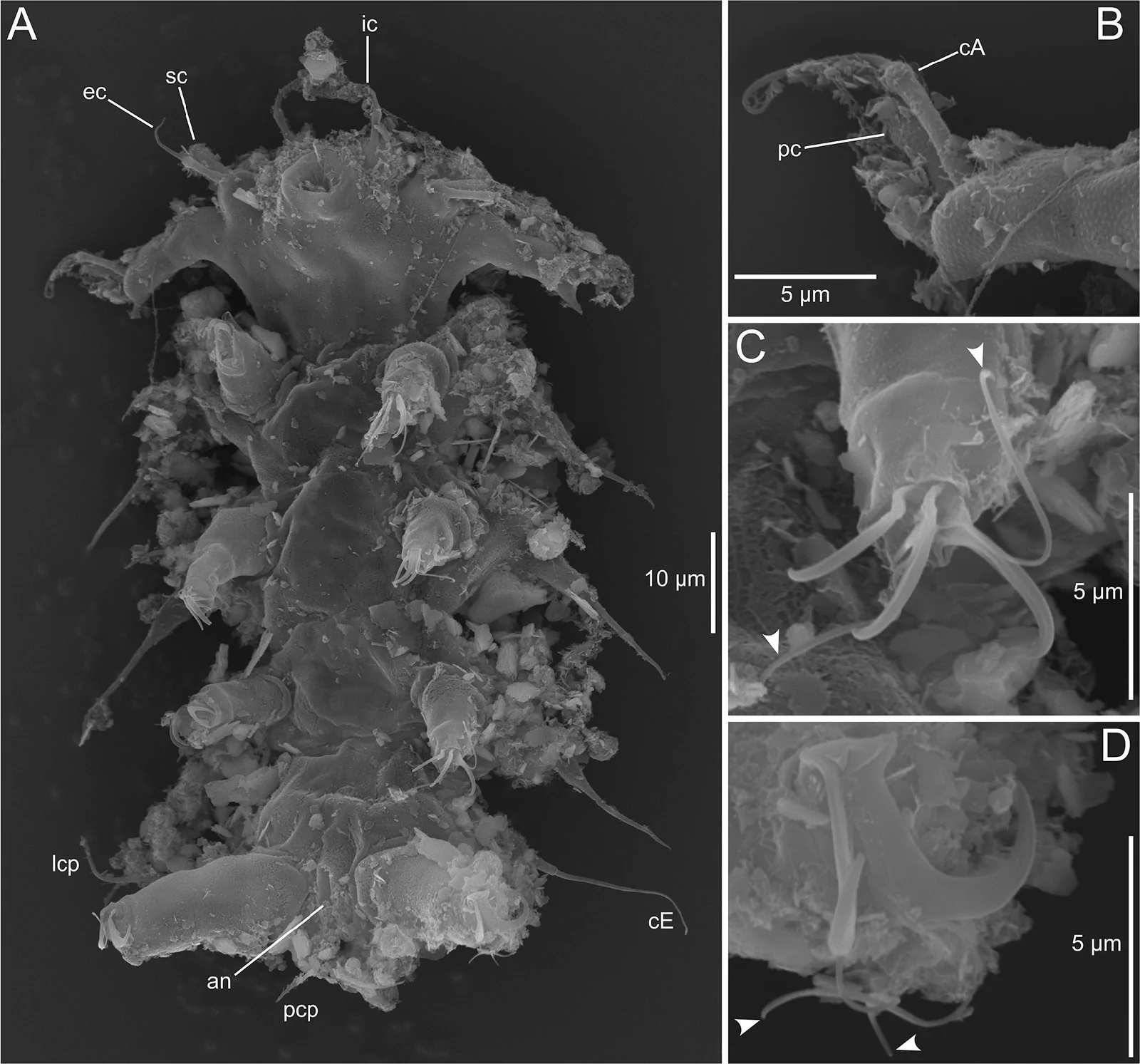
SEM micrographs of *Parastygarctus potercha* sp. nov. paratype juvenile (SMF 1483). **A**. habitus, ventral view; **B**. cephalic region, ventral view; **C**. leg III, note claw dorsal filament (arrowheads); **D**. leg IV, note claw dorsal filament (arrowheads). Abbreviations: an—anus, cA—cirrus *A*, cE—cirrus *E*, ec—external cirrus, ic—internal cirrus, lcp—lateral caudal process, pc—primary clava, pcp—posterior caudal process, sc—secondary clava.

Figs 7, 8, 9, 10, 11, Table 4

###### Diagnosis.

*Parastygarctus* with three body plates, each with two pairs of lateral acute processes: short and ventral anterior, long and dorsal posterior. Caudal plate with three pairs of processes: a pair of lateral spine-like processes, a pair of dorsolateral processes bearing cirri E, and a pair of spine-like posterior processes. Primary and secondary clavae oval and pedunculated. The first three pairs of legs terminate with three claws: outermost and middle claws with dorsal filaments. Leg IV with two claws bearing dorsal filaments. Female seminal receptacles as small vesicles with short sinuous ducts.

**Table 4.** *Parastygarctus potercha* sp. nov. measurements of selected morphological structures. Numbers are given in micrometers (µm). Abbreviations: Min—minimum value, na—data not available, SD—standard deviation.

	**Holotype**	**Paratype**	**Paratype**	**Paratype**	**Paratype**	**Paratype**	**Paratype**	**Paratype**	**Paratype**	**Paratype**	**Paratype**	**Paratype**	**Mean**	**SD**	**Min**
**specimen**	**slide**	**slide**	**slide**	**slide**	**slide**	**slide**	**digital**	**slide**	**SEM stub**	**slide**	**slide**	**slide**			
**code**	**SMF 1493**	**SMF 1494**	**SMF 1427**	**SMF 1490**	**SMF 1496**	**SMF 1541**	**SMF 1604**	**SMF 1453**	**SMF 1483**	**SMF 1491**	**SMF 1492**	**SMF 1495**			
**sex**	**female**	**female**	**male**	**male**	**undetermined**	**undetermined**	**undetermined**	**juvenile**	**juvenile**	**juvenile**	**juvenile**	**juvenile**			
Body length	69.0	70.0	85.3	89.3	65.0	52.5	78.0	64.0	76.7	74.0	69.0	52.7	70,5	11,3	52,5
Body width	21.0	18.0	22.3	22.3	na	na	na	17.3	20.3	18.0	17.5	14.0	19,0	2,7	14
Median cirrus	8.5	9.3	9.0	9.0	6.7	10.0	8.0	na	10.8	6.0	10.0	10.0	8,8	1,5	6
Internal cirri	7.0	7.0	11.0	10.3	na	5.0	na	7.0	8.6	7.0	na	6.0	7,7	2,0	5
External cirri	10.5	9.7	9.5	9.0	6.7	6.0	na	11.0	10.9	8.3	7.0	7.3	8,7	1,8	6
Cirri *A*	9.8	13.0	13.0	11.8	7.5	7.8	na	9.6	12,7	8.3	9.6	11.0	10,4	2,1	7,5
Primary clavae	4.7	5.0	6.3	4.5	4.5	3.0	na	5.0	5.2	4.3	5.0	4.5	4,7	0,8	3
Secondary clavae	4.0	4.2	6.0	4.3	2.5	3.0	na	4.3	4.8	3.3	na	2.7	3,9	1,1	2,5
Cirri *E*	23.0	28.5	18.5	20.5	22.0	19.7	27.3	24.5	21.1	18.5	21.3	15.5	21,7	3,7	15,5
Leg IV sensory organ	1.7	2.0	2.3	2.0	1.5	1.5	na	na	na	1.5	1.5	1.5	1,7	0,3	1,5
Leg I claws	5.5	4.7	6.3	6.8	5.2	5.3	na	6.3	5.5	4.5	na	5.2	5,5	0,7	4,5
Leg II claws	4.5	4.3	5.3	5.0	5.0	4.7	na	na	5.5	4.0	na	4.3	4,7	0,5	4
Leg III claws	4.0	4.2	4.3	5.0	5.0	4.7	na	4.6	5.6	3.3	3.7	4.5	4,4	0,6	3,3
Leg IV claws	6.5	7.5	7.0	8.0	5.8	6.7	na	4.5	7.0	5.0	5.7	6.5	6,4	1,0	4,5
Anus–gonopore	6.0	5.7	2.5	2.5	na	na	na	na	na	na	2.0	na	3,7	1,9	2

###### Type locality.

Station 850, 17°11.31'N, 21°57.20'W, 102.4 m b.s.l., Senghor Seamount, summit central, September 2009, biogenic coarse sand, MUC sample. Paratypes were collected from stations 848, 849 and 850, from 101.3 m, 102.0 m, and 102.4 m b.s.l. respectively, Senghor Seamount, summit central, September 2009, biogenic coarse sand, MUC samples.

###### Type material.

***Holotype***: • 1♀ (M79-3-850-2_13v74, SMF 1493) on slide. ***Paratypes***: • 1♀ (M79-3-850-2_14v74, SMF 1494) on slide; • 2♂♂ (M79-3-848-5_1v26, SMF 1427; M79-3-850-2_10v74, SMF 1490) on slides; • 1 juvenile (M79-3-850-2_2v74, SMF 1483) prepared for SEM, on stub; • 4 juveniles (M79-3-849-2_1v35, SMF 1453; M79-3-850-2_11v74, SMF 1491; M79-3-850-2_12v74, SMF 1492; M79-3-850-2_15v74, SMF 1495) on slides; • 2 undetermined sex (M79-3-850-2_16v74, SMF 1496; M79-3-850-2_61v74, SMF 1541) on slides; • 1 undetermined sex (M79-3-850-2_3v74, SMF 1604), digital specimen.

###### Description.

***Holotype***. Mature female (69.0 µm long, 21.0 µm wide) with cuticular armour divided into cephalic plate, three body plates, two intersegmental plates and caudal plate. Cephalic plate divided into five lobes: one medial, two medio-lateral, and two lateral. Each lateral lobe bears a posterior spine (Fig. 8A). Elbows (antero-lateral lobate processes of lateral lobes) are slightly prominent. Each of the three body plates has two pairs of spine-like lateral processes: a small anterior, arising ventrally, and a large posterior, arising dorsally (Fig. 8A). Caudal plate has a pair of spine-like lateral processes (Fig. 8A), a pair of dorsolateral processes bearing cirri *E* (Fig. 8B), and a pair of spine-like posterior processes (Fig. 8E). Cuticle with fine punctation. All cephalic cirri consist of scapus with distal swelling and flagellum. Internal cirrus is 7.0 µm long. External cirrus is 10.5 µm long. Median cirrus (8.5 µm long) arises from a circular plate. Primary clava (4.7 µm long) consists of peduncle and oval papilla. Cirrus *A* (9.8 µm long, the left one has a broken flagellum) inserts separately from primary clava. Secondary clava (4.0 µm long) is similar in shape as primary clava (Fig. 8C). Mouth cone is retracted. The structures of bucco-pharyngeal apparatus are not visible. Cirrus *E* (23.0 µm long) arises from lateral process and consists of smooth proximal portion, annulated scapus and flagellum (Fig. 8B). Sensory organs only on legs IV. Leg IV sensory organ (1.7 µm long) as spherical papilla arising on short peduncle. The first three pairs of legs bear only three claws (5.5 µm, 4.5 µm, and 4.0 µm for first, second, and third pairs respectively), with outermost and middle having dorsal filaments. Leg IV bears two claws, which are the biggest and the widest (6.5 µm long) and have dorsal filaments. Small rosette-shaped female gonopore (Fig. 8E). Seminal receptacles as small vesicles (Fig. 8F, G). Seminal ducts are sinuous and short. Anus closed by two large lateral lobes and small anterior lobe (Fig. 8D). Anus is 6.0 µm posteriorly to gonopore. The holotype exhibits paedomorphic patterns in having a small body size and a low number of claws on both legs I–III (only three claws per limb) and legs IV (only two claws per limb), but at the same time possessing a gonopore and seminal receptacles.

###### Remarks on paratypes.

In total, 11 paratypes were investigated. The paratypes correspond to the morphology of the holotype (Figs 9, 10, 11). Only a weak sexual dimorphism is observed. Males are slightly larger and have a shorter anus–gonopore distance of approximately 4.0 µm (Fig. 10D). Pharyngeal bulb locates in the level between first and second leg pairs in male specimens (Figs 9, 10B), however, there is no information on female specimens. Male gonopore formed a broad tube with oval opening (Fig. 10D). During SEM examination of a juvenile specimen (Fig. 11) we were able to better examine cephalic structure and claws.

###### Differential diagnosis.

The presence of two pairs of lateral spines of the body plates is shared by *Parastygarctus higginsi* Renaud-Debyser, 1965, *P. biungulatus* Morone De Lucia, Grimaldi de Zio & D’Addabbo Gallo, 1984, *P. svennevigi* Hansen, Kristensen & Jørgensen, 2012, and *P. mediterranicus* D’Addabbo Gallo, De Zio Grimaldi & Sandulli, 2001. *Parastygarctus potercha* sp. nov. can easily be distinguished from all congeneric species by the unique claw formula having only three claws per leg on leg pair I–III and two claws per leg on leg pair IV. *Parastygarctus potercha* sp. nov. and *P. biungulatus* both have three pairs of caudal plate processes. However, in *P. potercha* sp. nov. the dorsolateral processes bear the cirri *E* whereas in *P. biungulatus* cirri *E* attach directly to the caudal plate. Furthermore, the posterior spine-like processes are much shorter and thinner in *P. potercha* sp. nov. compared to those of *P. biungulatus*.

###### Etymology.

*Potercha*, plural *poterchata* (Latinised from Ukrainian "*потерча", "потерчата"*) is the ghost of a dead, unbaptised child, unique to Ukrainian folklore. The species name refers to the paedomorphic appearance of this species.

## Discussion

### Tardigrada on seamounts

This is the first investigation of the fauna of marine tardigrades of SSM. The current study focuses on the biogenic sediments from the summit plateau of this Guyot of the Cape Verde archipelago. Its tardigrade community is species-rich compared to the investigated abyssal reference site. It consists of 20 marine tardigrade taxa: *Batillipes* sp., Halechiniscidae sp., Halechiniscidae gen. A sp. A., *Chrysoarctus briandi*, *Chrysoarctus* sp., *Dipodarctus* sp., *Halechiniscus* sp., *Halechiniscus cf. perfectus*, *Quisarctus armatus*, *Megastygarctides hekatae* sp. nov., *Parastygarctus potercha* sp. nov., Styraconyxidae sp., *Raiarctus colurus*, *Raiarctus* sp., Tholoarctus cf. natans natans, and five possibly new species of Florarctinae, *Dipodarctus aff. subterraneus*, *Angursa* sp., *Raiarctus aff. variabilis*, and *Tanarctus* sp. The deep-sea reference site, characterised by fine ooze sediments, exhibits only low abundance and low diversity of tardigrades, with only two species present, *Angursa abyssalis* and *Angursa* sp. However, given the unequal sample sizes, it is not possible to draw statistically significant conclusions from this observation.

So far, there is only a handful of other studies investigating the tardigrade communities of seamounts, such as Faroe Bank (Jørgensen and Kristensen 2001; Hansen et al. 2001, 2012; Hansen 2005, 2007; Hansen and Kristensen 2021), Condor Seamount (Kristensen et al. 2015), Great Meteor Seamount (Tchesunov 2018), and Princess Alice Bank (Trokhymchuk et al. 2025). While only four tardigrade taxa have so far been recorded from the Azorean Princess Alice Bank (Trokhymchuk et al. 2025), the well-studied Faroe Bank harbours 35 recorded tardigrade species (Jørgensen and Kristensen 2001; Hansen et al. 2001; Hansen 2005, 2007), indicating a tardigrade community of similar diversity to that of SSM. At the current state of knowledge, it is not possible to draw any pattern from this, such as a latitudinal (climatological, i.e., sub-arctic to tropic) gradient or correlation with depth or sediment parameters, although such effects may occur (Kieneke et al. 2025). The numbers of analysed samples are still very low and may bias the recorded diversity. It has to be stressed, though, that the number of analysed samples of the Princess Alice Bank compared to SSM is quite balanced with five to seven.

Overall, the rather high species counts observed on the Faroe Bank and SSM compared to reported deep-sea records in the Atlantic region (e.g., Gomes-Júnior et al. 2020; Trokhymchuk and Kieneke 2024, 2025) may support the hypothesis of seamounts as biodiversity hotspots for marine tardigrades. However, such conclusions should be treated with caution because deep-sea tardigrade communities remain understudied. As Trokhymchuk et al. (2024) highlighted, the deep sea may host a broader diversity of tardigrades than currently recognised, extending traditionally reported deep-sea genera *Angursa* and *Coronarctus* (e.g., Kaczmarek et al. 2015). Thus, the differences in diversity and endemism between seamounts and deep-sea plains may reflect unevenness in sampling rather than true ecological patterns.

### Remarks on *Megastygarctides hekatae* sp. nov.

Among all species of the genus *Megastygarctides*, *M. hekatae* sp. nov. and *M. christinae* show the highest morphological similarity; however, in addition to external morphological differences, they also differ in the morphology and anatomy of the anogenital system. The structure of the reproductive system is an important species-specific characteristic for marine armoured tardigrades (Hansen and Kristensen 2006), so we consider these trait differences as significant. These two closely related species probably represent sister taxa of one tropical lineage; *M. christinae* is reported for Arabian (Persian) Gulf (Hansen and Kristensen 2006). *Megastygarctides hekatae* sp. nov. is the deepest recorded species in the genus so far (Bartels et al. 2015; Kaczmarek et al. 2015).

### Remarks on paedomorphosis

Among all the *Parastygarctus* species, *P. potercha* sp. nov. and *P. biungulatus* are the only two species that exhibit paedomorphic characteristics, including small body size and legs’ I–IV reduced claw numbers in legs I–IV compared to all other species. Paedomorphosis in marine tardigrades was discussed by Hansen et al. (2012) and suggested for *Faroestygarctus dezioae* Hansen, Kristensen & Jørgensen, 2012, *Parastygarctus biungulatus* and *Pseudostygarctus galloae* Hansen, Kristensen & Jørgensen, 2012. The adults of these species all exhibit the larval character of having only two claws on all legs. Having three claws per leg on leg pair I–III and two claws per leg on leg pair IV, *P. potercha* sp. nov. is not the most obvious case of paedomorphosis. However, we believe that the smaller body size and subtle seminal receptacles, compared to congeneric species, are further supporting evidence of paedomorphosis.

### Determination of species

Since two new species are described in the course of this study, we present updated dichotomous keys for *Megastygarctides* (from Ürkmez et al. 2017 with changes), and for *Parastygarctus* (from Grimaldi de Zio et al. 1987 with changes). The genus *Megastygarctides* is now represented by seven species of armoured marine tardigrades.

### Key to species of the genus *Megastygarctides*, following the pictorial key (Fig. 12)

**Table:** 

1	Deep median anterior indentation of the head present	**2**
–	Deep median anterior indentation of the head absent	**4**
2	Body plates absent	*M. gerdae* Hansen & Kristensen, 2006 (Fig. 12a, a2)
–	Body plates present	**3**
3	Unicellular glands present as internal globules inside of the body, primary clavae balloon-shaped	***M. isounguis* Renaud-Mornant, 1981 (Fig. 12b)**
–	Unicellular glands absent, primary clavae club-shaped	***M. orbiculatus* McKirdy, Schmidt & McGinty-Bayly, 1976 (Fig. 12c)**
4	Epicuticular fibrils present, more developed on hind legs	**5**
–	Epicuticular fibrils absent	**6**
5	Three lateral projections with fibrils absent; a clump of fibrils on the legs, near the primary and secondary clavae and near the gonopore absent	***M. setoloso* Morgan & O’Reilly, 1989 (Fig. 12d)**
–	Three lateral projections with fibrils present; a clump of fibrils on the legs, near the primary and secondary clavae and near the gonopore present	***M. sezginii* Ürkmez, Ostrowska, Roszkowska, Gawlak, Zawierucha, Kristensen & Kaczmarek, 2017 (Fig. 12e, e2)**
6	Primary clavae club-shaped, long genital spines, ovoid seminal receptacles	***M. christinae* Hansen & Kristensen, 2006 (Fig. 12f, f2)**
–	Primary clavae elongated, short genital spines, bean-shaped seminal receptacles	***M. hekatae* sp.nov. (Fig. 12g, g2)**

The genus *Parastygarctus* is now represented by eight species of armoured marine tardigrades.

**Figure 12.**
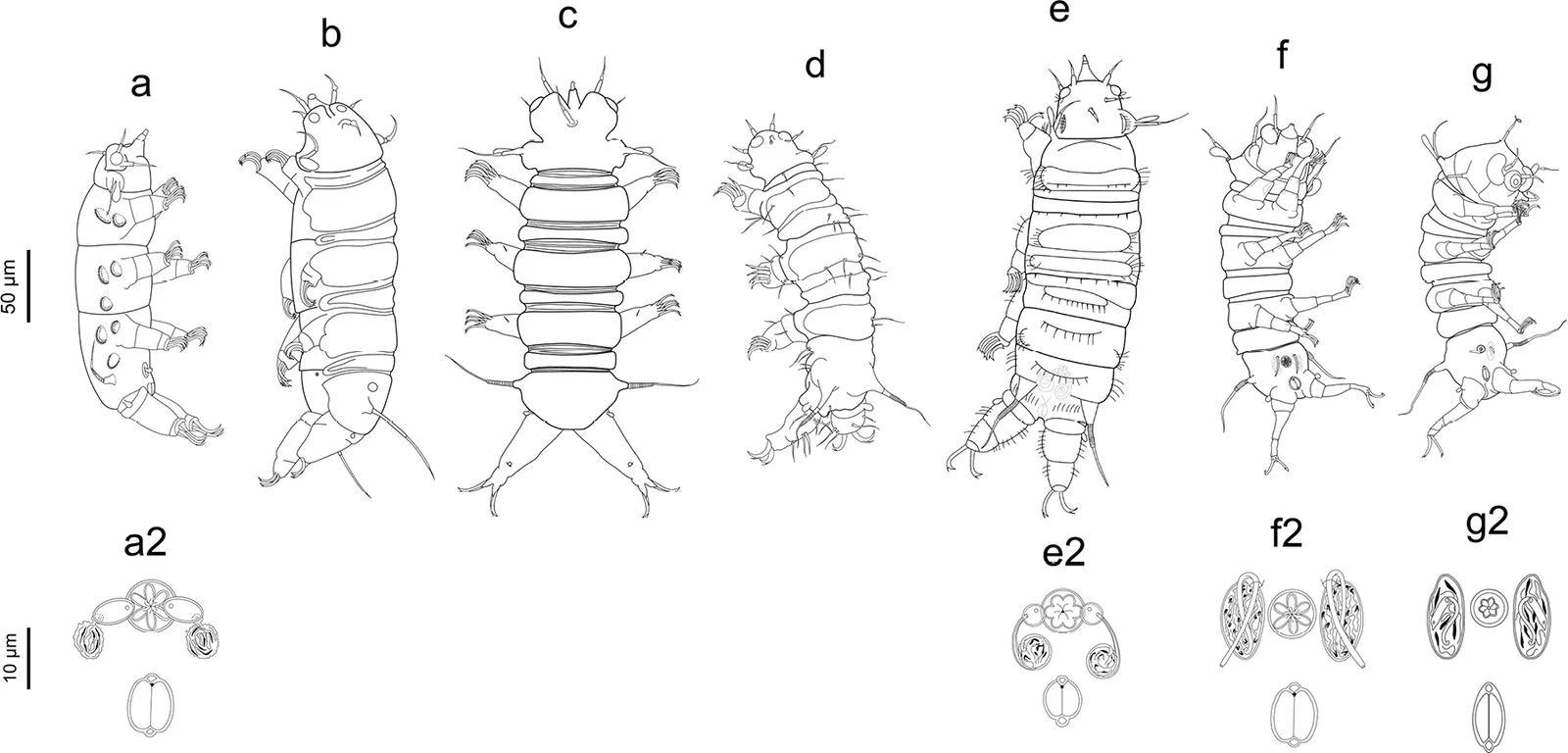
Pictorial key for genus *Megastygarctides*. **a**. *M. gerdae*, habitus; **a2**. *M. gerdae*, gonopore, seminal receptacles, and anus; **b**. *M. isounguis*, habitus; **c**. *M. orbiculatus*, habitus; **d**. *M. setoloso*, habitus; **e**. *M. sezginii*, habitus; **e2**. *M. sezginii*, gonopore, seminal receptacles, and anus; **f**. *M. christinae*, habitus; **f2***M. christinae*, gonopore, seminal receptacles, and anus; **g**. *M. hekatae* sp.nov., habitus; **g2**. *M. hekatae* sp.nov., gonopore, seminal receptacles, and anus. Redrawn after Hansen and Kristensen 2006 (Fig. 12a, a2); Renaud-Mornant 1981 (Fig. 12b); McKirdy et al. 1976 (Fig. 12c); Morgan and O’Reilly 1989; O’Reilly (1999) (Fig. 12d); Ürkmez et al. 2017 (Fig. 12e, e2); Hansen and Kristensen 2006 (Fig. 12f, f2).

### Key to species of the genus *Parastygarctus*, following the pictorial key (Fig. 13)

**Table:** 

1	Adult with fewer than four claws on legs I–III	**2**
–	Adult with four claws on legs I–III	**3**
2	Legs I–III with two claws, caudal plate with long posterior spines, cephalic cirri each with short scapus	***P. biungulatus* Morone De Lucia, Grimaldi de Zio & D’Addabbo Gallo, 1984 (Fig. 13a, a2)**
–	Legs I–III with three claws, caudal plate with short posterior spines, cephalic cirri each with long scapus	***P. potercha* sp. nov. (Fig. 13b, b2)**
3	Body plates with two lateral expansions	**4**
–	Body plates with one lateral expansion	**6**
4	Cephalic plate with deep anteromedial indentation, cephalic cirri each with long scapus, caudal plate with single lateral process	***P. higginsi* Renaud-Debyser, 1965 (Fig. 13c, c2)**
–	Cephalic cirri each with short scapus, caudal plate with two lateral processes	**5**
5	Caudal plate with long posterolateral process, elongated club-shaped clavae	***P. svennevigi* Hansen, Kristensen & Jørgensen, 2012 (Fig. 13d, d2)**
–	Caudal plate with short posterolateral process, short club-shaped clavae	***P. mediterranicus* Gallo D’Addabbo, Grimaldi de Zio & Sandulli, 2001 (Fig. 13e)**
6	Cephalic plate with lateral processes bent ventrally, clavae pedunculated	***P. renaudae* Grimaldi de Zio, D’Addabbo Gallo, Morone De Lucia & Daddabbo, 1987 (Fig. 13f, f2)**
–	Cephalic plate with straight lateral processes, clavae club-shaped	7
7	Median cirrus inserted in plate-like sculpture, highly looped seminal receptacle ducts, large seminal receptacle vesicles	***P. robustus* Hansen, Kristensen & Jørgensen, 2012 (Fig. 13g, g2)**
–	Seminal receptacle opens in funnel-shaped cavity	***P. sterreri* Renaud-Mornant, 1970 (Fig. 13h, h2)**

**Figure 13.**
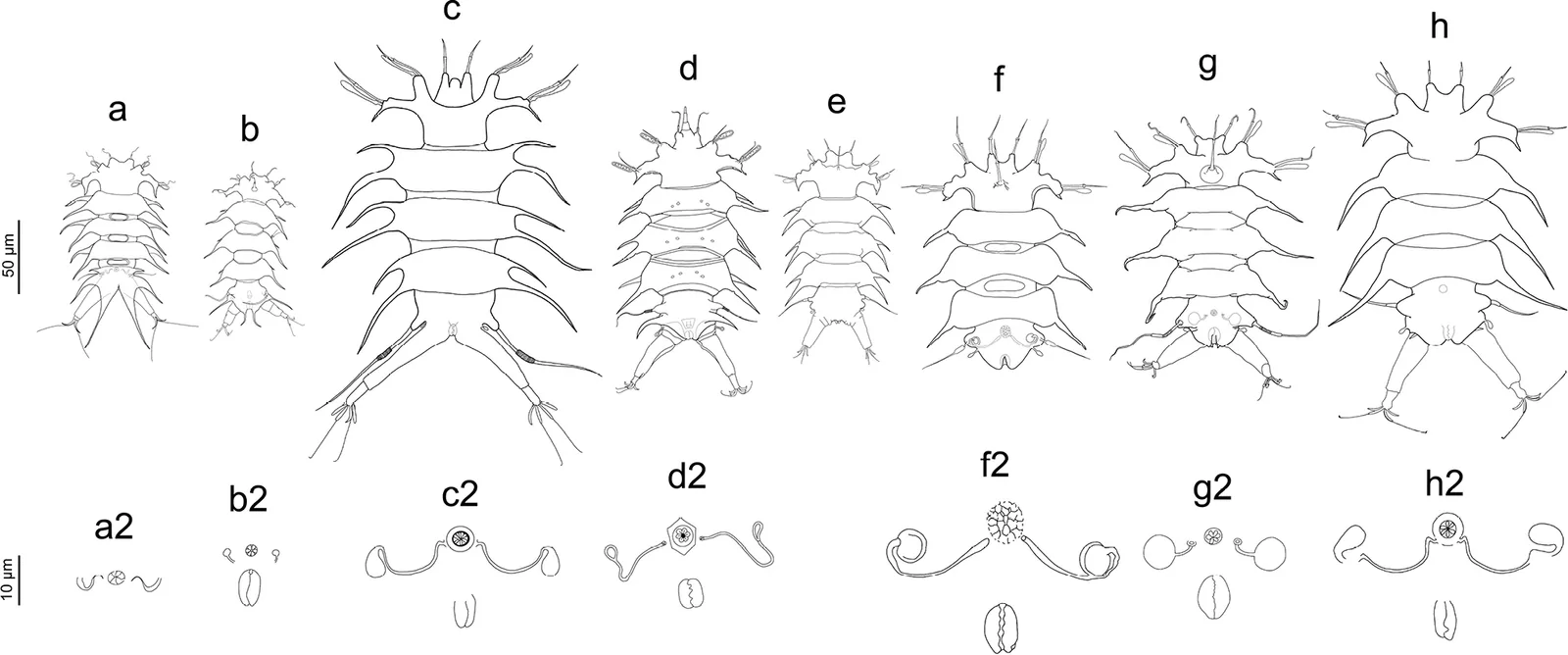
Pictorial key for genus *Parastygarctus*. **a**. *P. biungulatus*, habitus; **a2**. *P. biungulatus*, gonopore and seminal receptacles; **b**. *P. potercha* sp. nov., habitus; **b2**. *P. potercha* sp. nov., gonopore, seminal receptacles, and anus; **c**. *P. higginsi*, habitus; **c2**. *P. higginsi*, gonopore, seminal receptacles, and anus; **d**. *P. svennevigi*, habitus; **d2**. *P. svennevigi*, gonopore, seminal receptacles, and anus; **e**. *P. mediterranicus*, habitus; **f**. *P. renaudae*, habitus; **f2**. *P. renaudae*, gonopore, seminal receptacles, and anus; **g**. *P. robustus*, habitus; **g2**. *P. robustus*, gonopore, seminal receptacles, and anus; **h**. *P. sterreri*, habitus; **h2**. *P. sterreri*, gonopore, seminal receptacles, and anus. Redrawn after Morone De Lucia et al. 1984 (Fig. 13a, a2); Renaud-Debyser 1965 (Fig. 13c, c2); Hansen et al. 2012 (Fig. 13d, d2); D’Addabbo Gallo et al. 2001 (Fig. 13e); Grimaldi de Zio et al. 1987 (Fig. 13f, f2); Hansen et al. 2012 (Fig. 13g, g2); Renaud-Mornant 1970 (Fig. 13h, h2).

## Supplementary Material

XML Treatment for Megastygarctides hekatae

XML Treatment for Parastygarctus potercha

## Additional information

### Artificial Intelligence (AI) use

The authors accept full responsibility for the content of the manuscript, including the disclosure of any use of AI.

No AI tools were used in the preparation of this manuscript.

### Author ORCIDs

R.R. Trokhymchuk https://orcid.org/0000-0001-9570-0226

J.G. Hansen https://orcid.org/0000-0003-0054-0237

U. Pysarenko https://orcid.org/0009-0001-1775-5050

K. Subota https://orcid.org/0009-0001-3747-2373

A. Kieneke https://orcid.org/0000-0001-8180-2483

### Data availability

All of the data that support the findings of this study are available in the main text.
